# Outcomes of ventricular tachycardia ablation facilitated by pre-procedural cardiac imaging-derived scar characterization: a prospective multi-centre international registry

**DOI:** 10.1093/europace/euaf051

**Published:** 2025-03-14

**Authors:** Diego Penela, Giulio Falasconi, David Soto-Iglesias, Juan Fernández-Armenta, Giulio Zucchelli, Felipe Bisbal, Fatima Zaraket, Etelvino Silva, Matteo Parollo, Alessia Chiara Latini, Jose Alderete, Daniel Viveros, Aldo Bellido, Dario Turturiello, Chiara Valeriano, Paula Franco-Ocaña, Andrea Saglietto, Pietro Francia, Julio Martí-Almor, Antonio Berruezo

**Affiliations:** Arrhythmology Department, IRCCS Humanitas Research Hospital, Rozzano, Italy; Arrhythmology Department, IRCCS Humanitas Research Hospital, Rozzano, Italy; Heart Institute, Teknon Medical Centre, C/ Vilana, 12, 08022, Barcelona, Spain; Department of Cardiology, Puerta del Mar University Hospital, Cadiz, Spain; Cardiac Thoracic and Vascular Department, University Hospital of Pisa, Pisa, Italy; Heart Institute, Germans Trias i Pujol University Hospital, Badalona, Spain; Heart Institute, Teknon Medical Centre, C/ Vilana, 12, 08022, Barcelona, Spain; Department of Cardiology, Puerta del Mar University Hospital, Cadiz, Spain; Cardiac Thoracic and Vascular Department, University Hospital of Pisa, Pisa, Italy; Arrhythmology Department, IRCCS Humanitas Research Hospital, Rozzano, Italy; Department of Biomedical Sciences, Humanitas University, Pieve Emanuele, Milan, Italy; Heart Institute, Teknon Medical Centre, C/ Vilana, 12, 08022, Barcelona, Spain; Heart Institute, Teknon Medical Centre, C/ Vilana, 12, 08022, Barcelona, Spain; Heart Institute, Teknon Medical Centre, C/ Vilana, 12, 08022, Barcelona, Spain; Heart Institute, Teknon Medical Centre, C/ Vilana, 12, 08022, Barcelona, Spain; Heart Institute, Teknon Medical Centre, C/ Vilana, 12, 08022, Barcelona, Spain; Heart Institute, Teknon Medical Centre, C/ Vilana, 12, 08022, Barcelona, Spain; Heart Institute, Teknon Medical Centre, C/ Vilana, 12, 08022, Barcelona, Spain; Department of Medical Sciences, University of Turin, Turin, Italy; Division of Cardiology, Cardiovascular and Thoracic Department, ‘Città della Salute e della Scienza’ Hospital, Turin, Italy; Heart Institute, Teknon Medical Centre, C/ Vilana, 12, 08022, Barcelona, Spain; Division of Cardiology, Department of Clinical and Molecular Medicine, St Andrea Hospital, Sapienza University, Rome, Italy; Heart Institute, Teknon Medical Centre, C/ Vilana, 12, 08022, Barcelona, Spain; Heart Institute, Teknon Medical Centre, C/ Vilana, 12, 08022, Barcelona, Spain

**Keywords:** Catheter ablation, Cardiac imaging, Structural heart disease, Ventricular tachycardia, Substrate ablation, Multi-detector computed tomography, Cardiac magnetic resonance

## Abstract

**Aims:**

Pre-procedural imaging can facilitate scar-related ventricular tachycardia (VT) ablation, although only limited data have been reported. This prospective registry aimed to analyse procedural data and outcomes in a multi-centre setting of a pre-defined VT ablation strategy facilitated by the integration of pre-procedural imaging into the navigation system.

**Methods and results:**

Consecutive patients referred for scar-related left-sided VT ablation were prospectively enrolled at five European tertiary hospitals. Pre-procedural cardiac magnetic resonance (CMR)–derived scar maps and/or multi-detector computed tomography (MDCT)–derived wall thinning maps of the left ventricle (LV) were obtained and integrated into the navigation system. An endocardial or endoepicardial approach was chosen based on the scar distribution at pre-procedural imaging. The decision of performing a detailed electro-anatomical map (EAM) of the LV (image-aided) or to using the pre-procedural imaging for guiding the ablation without obtaining an EAM (image-guided) was left to the physician’s discretion. One hundred and seventy-one patients (71% with ischaemic cardiomyopathy) were included. Cardiac magnetic resonance was integrated in 159 (93%), MDCT in 113 (66%), and both in 101 (59%) procedures. Procedure-related complications occurred in 9 (5%) patients. At a mean follow-up of 18 ± 19 months, the overall survival and VT recurrence-free survival were 91 and 74.4%, respectively. There were no significant differences in long-term ablation outcomes based on the type of cardiomyopathy (*P* = 0.88) or the pre-procedural imaging modality employed (*P* = 0.33). An image-guided approach appears feasible, safe, and faster, with reduced procedure, radiofrequency, and fluoroscopy times, without compromising efficacy.

**Conclusion:**

In a large multi-centre prospective cohort, VT ablation facilitated by pre-procedural imaging is associated with favourable long-term outcomes.

## Graphical Abstract

**Graphical Abstract:**
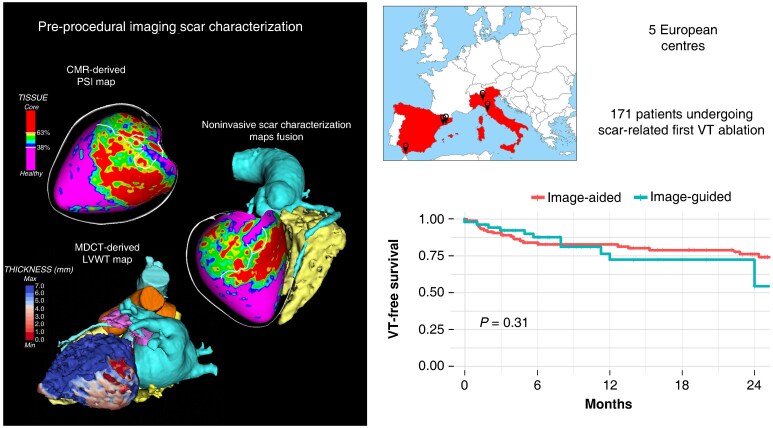
CMR, cardiac magnetic resonance; LVWT, left ventricle wall thickness; MDCT, multi-detector computed tomography; PSI, pixel signal intensity; VT, ventricular tachycardia.

**What’s new?**

- This is a large multi-centre prospective registry conducted across five centres with different levels of expertise, validating a pre-defined ventricular tachycardia ablation strategy that incorporates pre-procedural cardiac imaging integration within the navigation system. This approach was associated with favourable outcomes, with the overall survival and arrhythmia recurrence-free survival rates of 91 and 74.4%, respectively, at a mean follow-up of 18 ± 19 months.
- Ventricular tachycardia ablation facilitated by pre-procedural cardiac imaging narrows the gap in ventricular tachycardia-free survival between patients with ischaemic and non-ischaemic cardiomyopathy.
- An image-guided approach using the pre-procedural cardiac imaging for selecting the ablation target without obtaining an electro-anatomical map appears feasible and safe. This approach was associated with lower procedure, radiofrequency, and fluoroscopy times, without compromising efficacy.

## Introduction

Catheter ablation is an established and effective strategy for the treatment of ventricular tachycardias (VTs) in patients with structural heart disease (SHD).^1–4^ However, recurrence rate is not negligible, especially in the subgroup of patients with non-ischaemic cardiomyopathy (NICM).^5^ Myocardial re-entry is the main underlying mechanism of VTs in patients with SHD, and the principal arrhythmogenic substrate is the presence of viable strands of myocardium within zones of dense fibrosis that could generate re-entry circuits.^6^ This substrate can be identified and effectively targeted during stable sinus or paced rhythm, permitting the abolishment of multiple VT channels irrespective of the VT inducibility or haemodynamic tolerability and reporting a low complication rate.^3^ Pre-procedural cardiac imaging proved to be useful for the selection of the procedural approach^7,8^ and to permit focusing the map in the area of interest.^9^ Beyond, recent studies demonstrated that colour-coded pixel signal intensity (PSI) maps derived from pre-procedural cardiac magnetic resonance (CMR) and colour-coded left ventricle (LV) wall thinning (LVWT) maps derived from pre-procedural multi-detector computed tomography (MDCT) allow to characterize the scar tissue, facilitating VT ablation procedures.^10,11^ Pre-procedural non-invasive scar characterization (NSC) derived from both methods (CMR and/or MDCT) proved to correlate with the low-voltage areas and the distribution of local abnormal electrical activities. Initial data reported that an image-aided approach improves recurrence-free survival and procedural safety, and reduces procedural requirements in terms of skin-to-skin procedure time, radiofrequency (RF) delivery, and fluoroscopy dose.^10,11^ To date, only limited data are available regarding the safety and long-term efficacy outcomes of VT ablation facilitated by the integration of pre-procedural imaging into the navigation system. As a consequence, even if the current expert consensus^12,13^ strongly recommends the use of pre-procedural imaging for the ablation planning, the NSC integration into the navigation system in order to aid VT ablation procedure is only used in few specialized centres.^14^ Moreover, an approach aimed for a totally image-guided selection of the ablation target was recently described;^15,16^ however, the procedural feasibility and the results presented in this single-centre pilot study have not yet been corroborated in a multi-centre study.

The aim of this prospective registry is to analyse procedural data and outcomes in a multi-centre setting of a pre-defined VT ablation strategy facilitated by the integration of pre-procedural imaging into the navigation system.

## Methods

### Study design and patient sample

This multi-centre prospective study was conducted at five European tertiary hospitals. The study complied with the Declaration of Helsinki and was approved by the institutional review committees of the participating centres. All patients provided written informed consent.

Consecutive patients who underwent first VT ablation were prospectively enrolled. Sustained monomorphic (SM) VT was defined as any ventricular rhythm faster than 100 beats/min, lasting ≥30 s, or requiring termination due to haemodynamic instability by anti-tachycardia pacing or shocks. The inclusion criteria were: (i) at least one episode of symptomatic SMVT documented either by ECG or by implantable cardiac defibrillator (ICD) recording; (ii) indication for VT ablation in accordance to ESC guidelines;^17^ and (iii) diagnosis of SHD, including previous myocardial infarction, previous myocarditis, or dilated cardiomyopathy. In order to homogenize the characteristics of the patient sample, only left-sided scar-related VT ablations were included. The exclusion criteria were: (i) ventricular arrhythmias due to reversible causes; (ii) patients with arrhythmogenic right ventricular cardiomyopathy; (iii) contraindication to perform both CMR and MDCT; and (iv) contraindication to anti-coagulant drugs. In the absence of contraindications, both CMR and MDCT were performed before the VT ablation procedure.

### Pre-procedural cardiac magnetic resonance acquisition and post-processing

Pre-procedural CMR was obtained using a 1.5 T or a 3 T scanner. Contrast-enhanced images were acquired 10 min after bolus injection of 0.2 mmol/kg gadobutrol. In patients previously implanted with CMR-conditional ICD, a 1.5 T scanner and a specific wide-band sequence was used to avoid device-related artefacts (Inversion Recovery Turbo Field Echo IR-TFE, for Philips CMR research software R5.7). All CMR images were processed with ADAS-3D software (Galgo Medical, Barcelona, Spain) using a previously described protocol.^18^ Briefly, a full LV volume was reconstructed, and the resulting LV maps were divided into nine layers from the endocardium to the epicardium, obtaining a three-dimensional (3D) LV shell for each layer. Myocardial tissue was classified into scar core zone, border zone (BZ), and healthy tissue using 60 ± 5% and 40 ± 5% of the maximum PSI of the scar as thresholds. Left ventricle colour-coded PSI maps were obtained and projected to each of the shells following a trilinear interpolation algorithm. Border zone channels (BZCs) were defined as continuous corridors of BZ tissue connecting two areas of healthy tissue and surrounded by core scar or core scar and an anatomical barrier (e.g. mitral annulus). The scar mass, core scar mass, BZ mass, and BZC mass were automatically calculated by the software.

### Pre-procedural multi-detector computed tomography acquisition and post-processing

Pre-procedural MDCT was obtained using a 128-slice CT scanner. Images were acquired during an inspiratory breath-hold using retrospective ECG-gaiting technique with tube current modulation set between 50 and 100% of the cardiac cycle and during the injection of a 100 mL bolus of iopromide 370 mg/mL at a rate of 3 mL/s.

Multi-detector computed tomography images were analysed with ADAS-3D software, using a previously described protocol.^19^ Briefly, the endocardial and epicardial surfaces of the LV were automatically segmented using an AI-based algorithm, followed by manual corrections (see Supplementary material online methods for details). The LVWT was automatically computed by the software as the distance between each point on the endocardial and epicardial surfaces of the model and represented as a 3D colour-coded map. The scar area was coloured red and was defined as the area of LV thickness <5 mm; the rest of the map was coloured in various shades of red and blue based on local LVWT. Within the scar area, the software detected semi-automatically myocardial ridges, named computed tomography channels, defined as channel-shaped myocardial strands inside the scar area showing an LV thickness that exceeded the surrounding myocardium by ≥1 mm.^19^

**Figure 1:**
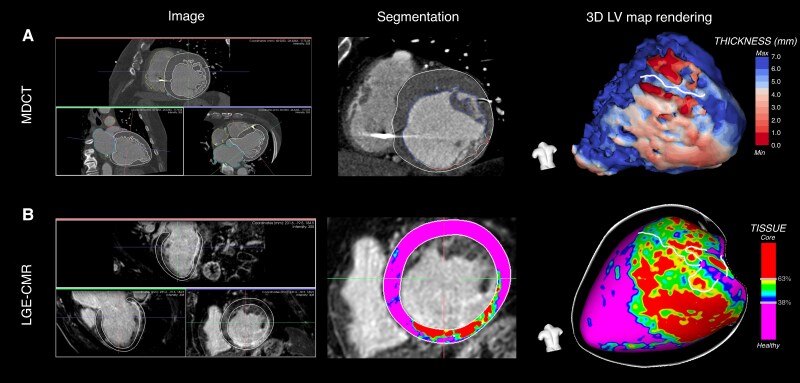
Cardiac magnetic resonance- and MDCT-derived data post-processing and NSC maps reconstruction of a post-myocardial infarction patient. (*A*) Pre-procedural MDCT-derived image data post-processing and 3D LV wall thinning map rendering with computed tomography channel detection in a patient with ischaemic cardiomyopathy who experienced an inferior myocardial infarction. (*B*) Pre-procedural CMR-derived image data post-processing and pixel signal intensity map rendering with BZCs detection in the same patient. LGE-CMR, late gadolinium enhancement-cardiac magnetic resonance; LV, left ventricle; MDCT, multi-detector computed tomography.

An example of CMR- and MDCT-derived data post-processing and NSC maps reconstruction of a post-myocardial infarction patient was represented in *Figure 1.*

### Procedural settings and image integration into the navigation system

Procedures were performed under general anaesthesia with haemodynamic monitoring using a radial arterial line. Peri-procedural anti-coagulation was performed according to local protocols, aiming to achieve an intraprocedural activated clotting time >300 s. A 3D navigation system and a 3.5 mm-tip open-irrigated ablation catheter were used in all the procedures while a multi-polar mapping catheter was used at the discretion of the operator.

**Figure 2:**
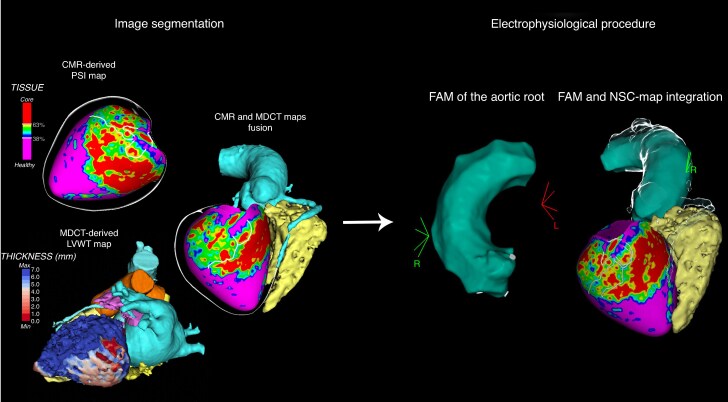
Workflow of NSC-derived map integration into the navigation system for ablation procedure. (*A*) Cardiac magnetic resonance-derived pixel signal intensity map with BZC detection and MDCT-derived LV wall thinning map with computed tomography channel detection superimposition according to standard reference points. (*B*) The first step of the ablation procedure was the acquisition of an FAM of the aorta in order to integrate MDCT and CMR-derived maps within the spatial reference coordinates of the navigation system. CMR, cardiac magnetic resonance; FAM, fast anatomical map; LVWT, left ventricle wall thinning; MDCT, multi-detector computed tomography; NSC, non-invasive scar characterization; PSI, pixel signal intensity.

An initial endocardial and/or epicardial approach was chosen according to CMR- and/or MDCT-derived information.^20,21^ The first step of the procedure was the acquisition of a fast anatomical map (FAM) of the ascending aorta, pulmonary arteries, or the left atrium at the discretion of the operator, in order to integrate the NSC-delivered maps within the spatial reference coordinates of the navigation system (*Figure 2*). For LV endocardial mapping, a transseptal approach was recommended by the study protocol, but the choice between transseptal and retro-aortic approaches was according to the operator’s choice.

### Ablation approach and procedure endpoints

**Figure 3:**
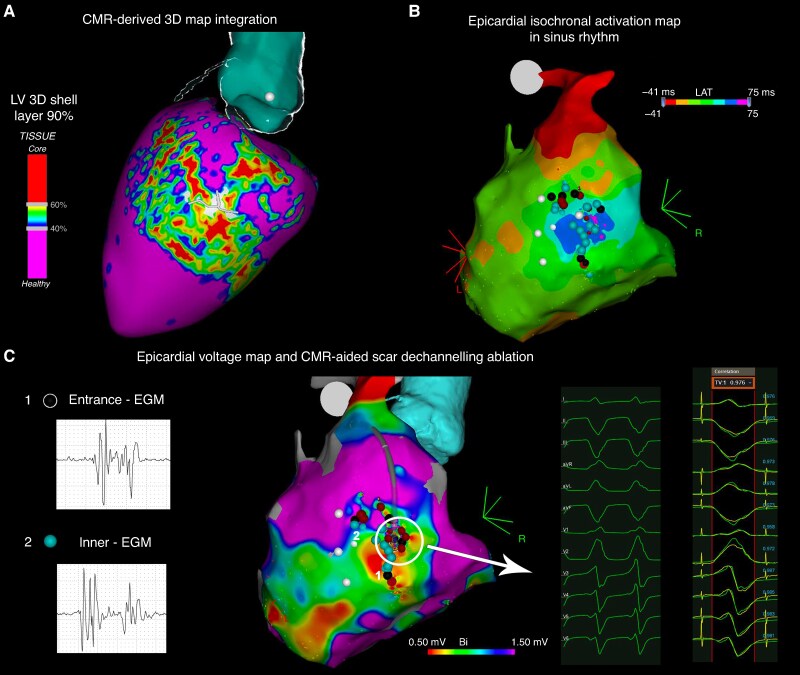
Cardiac magnetic resonance-aided epicardial VT ablation in a post-myocarditis patient. (*A*) Cardiac magnetic resonance-derived PSI map of LV epicardial layer with BZC detection integration into the navigation system. (*B*) Epicardial isochronal activation map during sinus rhythm and delayed EGM area identification. (*C*) Voltage EAM rendering and CMR-aided VT exit localization according to pacemapping manoeuvre followed by VT substrate ablation according to scar dechannelling technique, with entrance, and inner channel points RF ablation. CMR, cardiac magnetic resonance; EGM, electrogram; LV, left ventricle.

**Figure 4:**
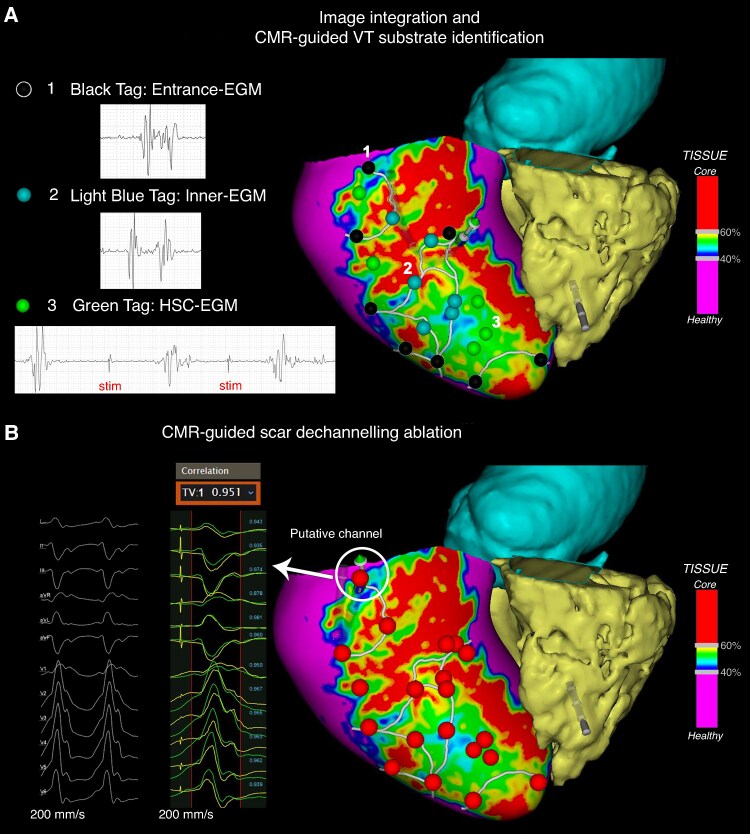
Cardiac magnetic resonance-guided endocardial VT ablation in a post-myocardial infarction patient with an inferior scar. (*A*) Cardiac magnetic resonance-guided localization of the arrhythmogenic substrate, classifying EGMs with delayed or fractionated components as entrance, inner, or HSC channel points depending on near-field precocity during sinus rhythm and post-RV extrastimuli. (*B*) Clinical VT QRS axis-based expected putative channel localization, and RF ablation; all the entrance and inner channel points and HSC-EGMs were targeted for ablation without the realization of an EAM of the LV. CMR, cardiac magnetic resonance; EGM, electrogram; VT, ventricular tachycardia.

**Figure 5:**
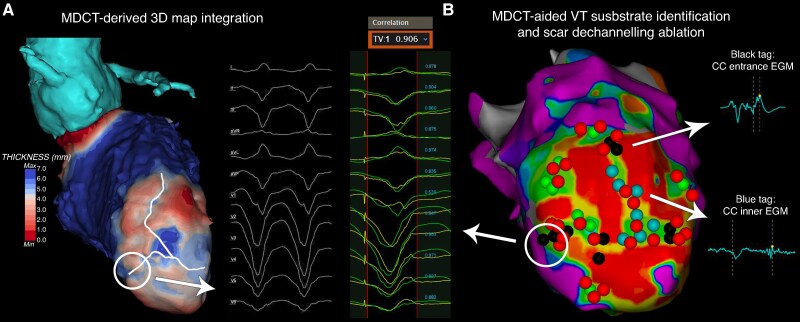
Multi-detector computed tomography-aided endocardial VT ablation in a post-myocardial infarction patient with an antero-apical scar. (*A*) Multi-detector computed tomography-derived LVWT map integration into the navigation system and imaging-aided VT exit localization according to pacemapping. (*B*) Voltage EAM rendering and VT substrate ablation according to scar dechannelling technique, with entrance, inner channel points, and HSC-EGMs ablation. CC, conducting channel; EGM, electrogram; MDCT, multi-detector computed tomography; VT, ventricular tachycardia.

After the initial mandatory step of integrating NSC-delivered maps into the navigation system, the choice to either perform a complete electro-anatomical map (EAM) focusing on the area of interest (image-aided approach^10,21–23^) or to select the ablation target directly from the NSC-derived maps (image-guided approach^14^) was left to the operator’s discretion, based on their prior experience and confidence with the technique. An exhaustive description of the ablation strategies is provided in the Supplementary material online. *Figures 3–5* show examples of CMR-aided, CMR-guided, and MDCT-aided ablations, respectively.

After VT substrate ablation, a programmed ventricular stimulation protocol from the right ventricle (RV) apex was performed in all patients to assess for inducible VTs. Any induced SMVT was targeted for ablation, either by activation mapping (if haemodynamically tolerated) or by pacemapping manoeuvres. Inducibility was checked again after each VT ablation. Acute procedural success was defined as non-inducibility of any SMVT at the end of the procedure. Partial acute procedural success was defined as non-inducibility of the clinical VT at the end of the procedure.

The primary safety endpoint was freedom from any procedure-related major adverse event (MAE) within 30 days of the procedure. The occurrence of any of the following was classified as MAE: cardiac tamponade, atrioventricular block, vascular injury requiring intervention, stroke, phrenic nerve palsy, acute heart failure, transient ST-segment elevation, and death.

### Post-procedural follow-up

All patients were discharged with an ICD, and the first VT zone with active therapies was programmed at 15 beats/min slower than the clinical VT heart rate. Clinical evaluation and ICD interrogation were scheduled every 6 months. Non-inducible patients after ablation did not receive any anti-arrhythmic treatment during the follow-up, excluding beta-blockers in case of clinical indication, such as heart failure, chronic ischaemic cardiomyopathy, or atrial fibrillation. The long-term primary endpoint was the VT recurrence-free survival. Any episode of SMVT or appropriate ICD therapy was considered VT recurrence.

### Statistical analysis

Continuous variables were presented as mean ± standard deviation or median (interquartile range) as appropriate. Categorical variables were presented as total number (percentage). The Student *t*-test or Wilcoxon test was used to compare continuous variables, as appropriate; χ^2^ or Fisher exact test was used to compare categorical variables, as appropriate. Kaplan–Meier curves and the log-rank test were used to assess the cumulative event-free survival. A level of *P* < 0.05 was considered for statistical significance. Data were analysed with R version 3.6.2 software (R Foundation for Statistical Computing, Vienna, Austria).

## Results

### Patient characteristics

A total of 171 consecutive patients with SHD undergoing a left-sided first VT ablation were included. The mean age was 66 ± 12 years, 159 (93%) patients were male, and 121 (71%) had an ischaemic cardiomyopathy (ICM) diagnosis. The mean left ventricular ejection fraction was 40 ± 12%. Included patients reported an average of 6 ± 2 VT episodes previous to the ablation procedure, while 46 (27%) patients experienced arrhythmic storm or incessant VT.

*Table 1* summarizes patients’ baseline characteristics according to the ablation approach.

**Table 1:** Patients’ baseline characteristics according to the ablation approach

	Image-aided (*n* = 108)	Image-guided (*n* = 63)	Total patients (*n* = 171)	*P*-value
Age (years)	66.0 ± 11.6	64.6 ± 13.4	65.5 ± 12.3	0.57
Male	99 (91.7)	60 (95.2)	159 (93.0)	0.54
Hypertension	73 (67.6)	36 (57.1)	109 (63.7)	0.19
Diabetes	23 (21.5)	18 (28.6)	41 (24.1)	0.35
Dyslipidaemia	67 (62.0)	33 (52.4)	100 (58.5)	0.26
COPD	14 (13.0)	6 (9.5)	20 (11.7)	0.60
CKD	14 (13.0)	9 (14.3)	23 (13.4)	0.80
LVEF (%)	40.1 ± 12.3	41.2 ± 11.8	40.5 ± 12.1	0.49
Atrial fibrillation	13 (12.0)	7 (11.1)	20 (11.7)	0.99
ICM	75 (69.4)	46 (73.0)	121 (70.8)	0.73
NICM diagnosis				0.26
Scarred non-dilated CM	17 (15.7)	6 (9.5)	23 (13.5)	
Dilated CM	6 (5.6)	7 (11.1)	13 (7.6)	
Other	10 (9.3)	4 (6.3)	14 (8.2)	
Pre-procedural ICD carriers	62 (57.4)	44 (69.8)	106 (62.0)	0.14
NYHA class	1.7 ± 0.7	1.7 ± 0.6	1.7 ± 0.6	0.63
β-Blockers	80 (74.1)	43 (68.3)	123 (71.9)	0.48
Class I AADs	1 (0.9)	3 (4.8)	4 (2.3)	0.14
Class III AADs	53 (49.1)	27 (42.9)	80 (46.8)	0.52
VT storm/incessant	33 (30.6)	14 (22.2)	47 (27.5)	0.29
Clinical VTCL (ms)	357.6 ± 98.2	360.8 ± 118.4	358.7 ± 104.8	0.85

AADs, anti-arrhythmic drugs; CKD, chronic kidney disease; CM, cardiomyopathy; COPD, chronic obstructive pulmonary disease; ICD, implantable cardiac defibrillator; ICM, ischaemic cardiomyopathy; VT, ventricular tachycardia; VTCL ventricular tachycardia cycle length.

### Pre-procedural cardiac imaging data

A pre-procedural CMR was obtained in 159 (93%) patients, using a 3 T scanner in 83 (52%) and a 1.5 T machine in 76 (48%). In 15 (9%) patients. a wide-band sequence was used. A pre-procedural MDTC was obtained in 113 (66%) patients, while 101 (59%) patients had both CMR and MDTC pre-procedural studies.

In 101 patients (59%), BZC maps from CMR were integrated into the navigation system along with MDCT information. In 58 patients (34%), only the BZC map from CMR was integrated, while in the remaining 12 patients (7%), only the MDCT-derived LVWT map was integrated.

### Procedural data

Transeptal puncture was the preferred approach for LV endocardial mapping (121 patients, 71%). Percutaneous pericardial access for epicardial mapping during the procedure was attempted in 50 (29%) patients, and successfully performed in 47 (94%) patients. A multi-polar mapping catheter was used in 51 (30%) patients. No LV assist device was used in any patient to aid the procedure.

The aortic root was the most frequently structure used for the image integration into the navigation system. The mean FAM acquisition time was 9.5 ± 2 min.

Only in the image-aided group, an EAM of LV was obtained; in this group, a mean of 1323 ± 825 points was collected in the endocardial EAM and 1060 ± 793 in the epicardial EAM. In the whole cohort, a mean of 11.0 ± 9.8 conducting channel entrance points and 35.3 ± 30.2 conducting channel inner points was identified per patient. When used, double extra-stimulus technique identified a median of 7.0 ± 6.0 hidden slow conduction (HSC)-electrogram per patient (see Supplementary material online for the definition).

### Acute procedural outcomes

A negative VT induction protocol after the substrate ablation was reported in 123 (72%) patients. A mean of 1.2 ± 0.4 residual VTs were induced in the remaining patients. Acute procedural success was obtained in 126 (74%) of patients. Clinical VT was eliminated in all but 11 (6%) patients. The mean procedure time was 192 ± 72 min with a mean RF time of 16 ± 11 min of RF delivery. The mean fluoroscopy time was 16 ± 10 min and a median of 25 ± 20 RF applications were delivered per patient. *Table 2* shows the procedural data in patients with ischaemic and non-ischaemic cardiomyopathy.

**Table 2:** Procedural data according to the cardiopathy type and to the ablation approach

	ICM (*n* = 121)	NICM (*n* = 50)	*P*-value	Image-aided (*n* = 108)	Image-guided (*n* = 63)	*P*-value	Total patients (*n* = 171)
Procedure time (min)	198 ± 78	178 ± 69	0.18	210 ± 69	159 ± 78	**< 0.001**	192 ± 72
Fluoroscopy time (min)	16 ± 10	16 ± 10	0.67	18 ± 10	13 ± 9	**0.003**	16 ± 10
RF time (min)	19 ± 11	10 ± 7	**<0.001**	18 ± 12	14 ± 9	**0.01**	17 ± 11
Epicardial mapping	30 (24.8)	17 (34.0)	0.26	34 (31.5)	13 (20.6)	0.3	47 (27.5)
Transeptal approach	101 (83.5)	20 (40.0)	**<0.001**	77 (71.3)	44 (69.8)	0.99	121 (70.8)
Multi-polar mapping catheter	44 (36.4)	7 (14.0)	**0.003**	34 (31.5)	17 (27.0)	0.61	51 (29.8)
VT non-inducibility post-first ablation attempt	88 (76.7)	35 (70.0)	0.71	69 (63.9)	54 (85.7)	**0**.**002**	123 (71.9)
MAEs	7 (5.8)	2 (4.0)	0.99	4 (3.7)	5 (7.9)	0.81	9 (5.3)
Incomplete substrate elimination	17 (14.0)	7 (14.0)	0.99	11 (10.2)	13 (20.6)	**0.04**	24 (14.0)
Acute procedural endpoints			0.5			0.2	
Acute procedural success	87 (71.9)	34 (68.0)		72 (66.7)	49 (77.8)		121 (70.8)
Partial procedural success	28 (23.1)	11 (22.0)		26 (24.1)	13 (20.6)		39 (22.8)
Unsuccessful	6 (5.0)	5 (10.0)		10 (9.3)	1 (1.6)		11 (6.4)

Bold values indicate *P*-values related to statistically significant differences.

ICM, ischaemic cardiomyopathy; MAE, major adverse event; NICM, non-ischaemic cardiomyopathy; RF, radiofrequency; VT, ventricular tachycardia.

Procedure-related complications occurred in nine (5%) patients: two patients had a vascular complication, two a complete AV block, two a phrenic nerve paralysis, one patient a peri-procedural cerebral ischaemic event, one patient a cardiac tamponade, and one patient suffered a myocardial perforation resolved by surgery. No procedural-related deaths occurred.

**Figure 6:**
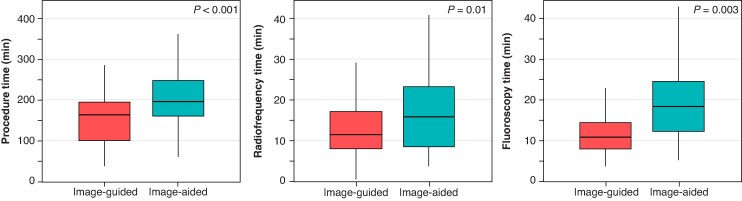
Procedural outcomes according to the ablation approach.

*Table 2* and *Figure 6* show the acute procedural data according to the ablation approach. The percentage of patients with a negative induction protocol after substrate ablation was higher in the image-guided approach compared with the image-aided group (86 vs. 61%, *P* = 0.002). However, there were no differences in the acute success rate at the end of the procedure between groups (*P* = 0.2). No difference was observed in the complication rate between groups. In the image-guided group, significantly shorter procedure time (159 ± 78 vs. 210 ± 69 min, *P* < 0.001), fluoroscopy time (12.8 ± 9 vs. 17.7 ± 10 min, *P* = 0.003), and RF time (13.6 ± 9 vs. 18.3 ± 12 min, *P* = 0.01) were observed. Finally, there was a trend towards a reduction in the number of RF applications in the image-guided group with respect to the image-aided group (26 ± 22 vs. 33 ± 21, *P* = 0.06).

### Outcomes

After a follow-up of 18 ± 19 months, the overall survival and VT recurrence-free survival were 91.0 and 74.4%, respectively. Supplementary material online, *Table S2* shows the predictors of VT recurrence.

**Figure 7:**
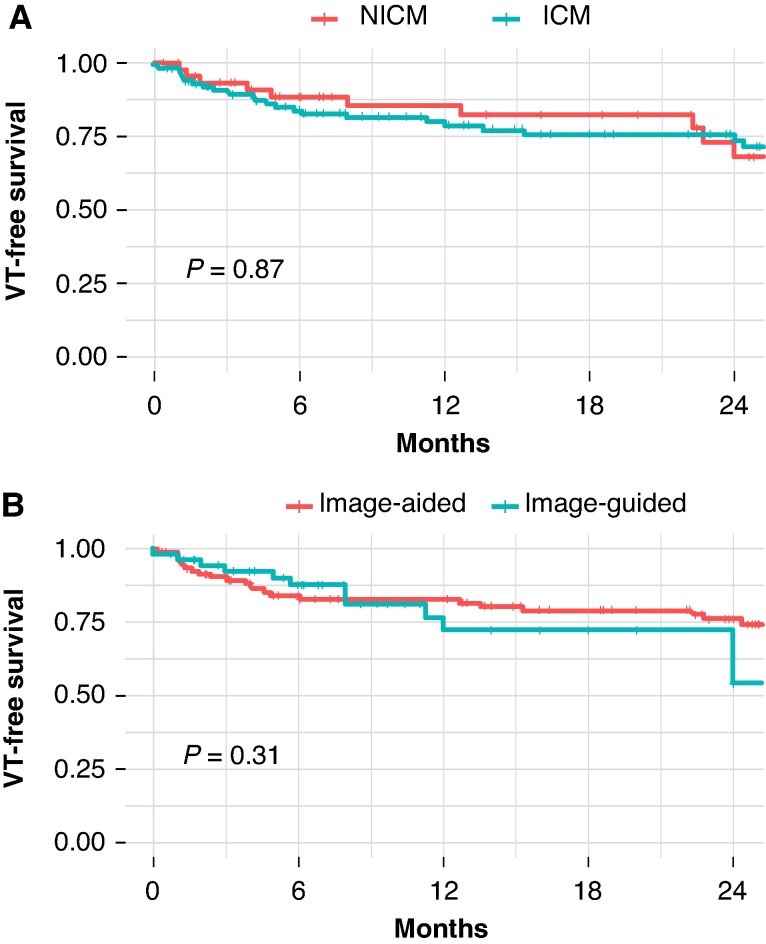
Ventricular tachycardia recurrence-free survival Kaplan–Meier curve according to the cardiomyopathy diagnosis (*A*) and according to the ablation approach (*B*). ICM, ischaemic cardiomyopathy; NICM, non-ischaemic cardiomyopathy; VT, ventricular tachycardia.

In the log-rank analysis, no significant differences were observed in the long-term ablation outcomes based on the cardiomyopathy type (ICM or NICM, *P* = 0.88), see *Figure 7* and Supplementary material online, *Figure S2*. Similarly, the VT-free survival did not differ according to the imaging modality used for pre-procedural scar characterization in the total population (MDCT, 1.5 T CMR, or 3 T CMR, *P* = 0.33) neither in ICD carriers (MDCT, 1.5 T CMR, Wideband, or 3 T CMR, *P* = 0.31), as shown in Supplementary material online, *Figure S1*.

Additionally, there were no significant differences in VT recurrence-free survival rates between the image-aided and image-guided approaches (*P* = 0.31). Finally, the VT recurrence rate did not vary significantly between different centres (*P* = 0.11).

*Figure 7* shows the VT recurrence-free survival Kaplan–Meier curves according to the cardiomyopathy diagnosis (*Figure 7A*) and according to the ablation approach (*Figure 7B*).

## Discussion

The main findings of the study are: (i) a VT ablation approach based on the integration in the navigation system of the pre-procedural image scar characterization results in favourable acute and long-term outcomes; (ii) this approach is also reproducible as no differences were found in outcomes between different centres with diverse degree of expertise; (iii) using pre-procedural imaging for selection of the correct approach and for delineating scar location and shape, seems to reduce the gap between ischaemic and non-ischaemic patients regarding VT-free survival; and (iv) selecting the ablation target based only in the imaging information, without performing an EAM, is feasible and safe. In the present multi-centre registry, this approach was associated with a reduction in the total procedure time.

### Pre-procedural image integration

Previous studies have showed that scar integration from imaging is an independent predictor of better VT-free survival after catheter ablation^22^ and reduces total procedure time.^23^ However, to the best of our knowledge, this is the first multi-centre registry reporting the results of a pre-defined protocol based on the integration of pre-procedural NSC, by means of CMR and/or MDCT, into the navigation system for helping VT ablation procedures. In this large multi-centre international prospective cohort, this strategy resulted in favourable long-term outcomes, with a 91% overall survival and a 25% recurrence rate at 18 months. It is worthy to underscore that after substrate ablation, 72% of patients were non-inducible for any SMVT, thus performing the entire procedure in sinus rhythm, which suggest a correct arrhythmogenic substrate identification and characterization by using this approach. These results are in line with a recent meta-analysis in which VT ablation aided by pre-procedural imaging results in a 94% overall survival rate and a recurrence rate ranging 20% in the mid-term, showing better outcomes when compared with conventional VT ablation.^24^  Supplementary material online, *Table S1* compares the outcomes observed in this registry with those of previous registers on substrate VT ablation. The favourable results of the present registry, including centres with different degrees of expertise, confirm that this approach is reproducible and can be replicated outside of specialized high-volume centres. Finally, the complication rate in the present series (5%) was inferior to that reported in the literature (8–10%).^12^

The favourable acute and long-term outcomes of this registry can be attributed to the theoretical advantages of using imaging to help the procedure: (i) first, imaging assists in selecting the appropriate access based on scar distribution,^9^ ensuring complete depiction to the arrhythmogenic substrate,^25^ while also minimizing risks (e.g. avoiding unnecessary epicardial punctures in patients who do not require them). (ii) Three-dimensional reconstruction through imaging provides accurate information about scar dimensions and shapes,^26^ in contrast to EAM which is often affected by the far-field effects of surrounding normal tissue (especially in highly heterogeneous scars). (iii) Imaging is capable of identifying arrhythmogenic substrate deep within the myocardial wall. In contrast, identifying mildly embedded arrhythmogenic substrate using EAM remains challenging. In this regard, Dickfeld *et al*.^27^ previously demonstrated that a viable endocardial layer of >2 mm covering the scar is sufficient to result in normal bipolar voltage readings in the EAM.

### Ischaemic vs. non-ischaemic cardiomyopathy

All of the above is particularly relevant for patients with NICM, who often present with more heterogeneous scars in uncommon locations (e.g. sub-epicardial, septal mid-wall distribution). Consistent with this challenging arrhythmia substrate, VT ablation in NICM patients has a lower likelihood of procedural success and an increased risk of ventricular arrhythmia recurrences compared with ischaemic patients. A recent systematic review^5^ indicates a 56% rate of acute procedural success and a recurrence rate of 26 per 100 patient-years in NICM patients who underwent VT ablation. Similarly, a meta-analysis involving 665 NICM patients reported a recurrence rate of 34% after the first ablation procedure.^28^ In this current registry, the acute procedural success and the recurrence did not differ significantly when compared with patients with ischaemic heart disease. These results could be attributed to a more accurate characterization of the arrhythmogenic substrate and improved substrate identification due to the systematic use of imaging to aid the procedure. Similarly, a previous study predominantly involving NICM with intramural scar achieved a 76% arrhythmia-free survival rate using an image-guided ablation approach.^29^ The favourable results observed with a pre-procedural imaging integration approach in NICM patients should be confirmed in controlled studies.

### Image-guided approach

Characterizing the arrhythmogenic substrate through EAM constitutes a crucial step in VT substrate ablation. Nevertheless, EAM is known to be time-consuming and demanding in terms of advanced catheter manoeuvring skills. Additionally, it is not without limitations (e.g. challenges in intramyocardial substrate characterization, difficulties in delineating the scar edge due to the far-field effect of normal myocardium).

A previous study involving 49 patients who underwent image integration-guided VT ablation at two highly experienced centres compared the outcomes of patients who had a complete EAM performed with those whose EAM was limited to the imaging-defined substrate. The latter approach was associated with shorter and more predictable procedure durations, achieving high long-term success rates.^23^ A previous pilot study conducted at a single centre introduced a comprehensive image-guided approach for VT ablation, foregoing the use of any EAM.^15^ In this pioneering study, VT ablation guided solely by imaging was both feasible and safe. Authors reported a significant reduction in procedural times and observed a low rate of VT recurrence following substrate ablation.

The findings of the current registry align with these earlier observations. Among patients who underwent the image-guided approach, the total procedure time was shorter, and there was a reduced need for fluoroscopy and RF time, all without compromising efficacy. These results validate this approach in a multi-centre setting, making the initial stride towards the dissemination of this methodology. However, this preliminary observation should be confirmed in randomized studies specifically designed for this purpose (NCT04694079).

### Study limitations

The registry lacks a comparison group using another ablation strategy. The present study was not designed to identify differences between the image-aided and image-guided approaches. Hence, we cannot exclude the possibility that the study may be underpowered to detect differences between groups. Moreover, the choice of approach for selecting the ablation target was ultimately at the discretion of the physician, potentially introducing a selection bias that cannot be ignored. A prospective randomized trial is currently underway to address this specific question (NCT04694079). The substrate ablation strategy was the same in all the patient population (scar dechannelling), and therefore, the outcomes cannot be extrapolated with certainty to other substrate ablation techniques.

Scar characterization on CMR was determined using the maximum PSI, applying the established thresholds of 60 ± 5% and 40 ± 5%.^17^ The potential impact of alternative scar characterization methods on these findings remains unclear.^30^

Finally, image post-processing did not include analyses of lipomatous metaplasia and epicardial fat distribution.

## Conclusions

In a large multi-centre prospective cohort, VT ablation facilitated by NSC-derived map integration into the navigation system is associated with good short- and long-term outcomes. No differences were observed in arrhythmia-free survival among centres with varying levels of expertise in image integration during VT ablation procedures. An image-guided approach appears feasible, safe, and faster, with reduced procedure, RF, and fluoroscopy times, without compromising efficacy.

## Supplementary Material

euaf051_Supplementary_Data

## Data Availability

The data that support the findings of this study are available from the corresponding author, upon reasonable request.
