# Supplementary material for: Outcomes of ventricular tachycardia ablation facilitated by pre-procedural cardiac imaging-derived scar characterization: a prospective multi-centre international registry
Source: Europace. 2025 Mar 14;27(4):euaf051. doi: 10.1093/europace/euaf051 (PMC11983391; doi:10.1093/europace/euaf051)
Supplement: euaf051_Supplementary_Data [file euaf051_supplementary_data.zip › Supplemental Methods.docx]

**Image-Aided and Image-Guided Approaches**

In the “image-aided” group, the CMR and/or MDCT-derived information were used for focusing the EAM of the LV scar area. The ablation targets were selected according to the EAM information([10](#_ENREF_10)). EGMs with delayed components (EGM-DCs) were tagged and dichotomously classified as entrance or inner points, depending on delayed-component precocity during sinus rhythm([21](#_ENREF_21)). EGMs with >3 deflections not meeting criteria for fractionated EGM (duration <133 ms) identified within the scar area or in the myocardium surrounding the scar area were considered potential hidden slow conduction (HSC) points: at these sites a double RV extrastimuli was delivered and response was considered positive when the local potential delayed and splitted from the far-field signal([22](#_ENREF_22)). According to the scar dechanneling technique,([23](#_ENREF_23)) each RF application lasted 30 to 60 seconds, aiming at the abolition of all identified entrances and positive HSC points. Backup RF applications were delivered inside the scar area when the RF delivery at the entrance points did not eliminate internal pathological EGMs into the scar area at the end of the ablation.

In the “image-guided” group, the EAM of the LV was not obtained, and PCI-maps were the only information used for selecting the ablation targets: RF was delivered at the entrances of CMR-derived BZCs and of the MDCT-derived CTCs. Local EGM information were only analyzed to avoid RF applications if EGMs presented His or fascicular characteristics, an amplitude >3mV, or between 1.5 and 3 mV without delayed components, as previously described ([14](#_ENREF_14)).

An exhaustive description of the ablation strategies is provided in the Supplementary Materials.

**Ablation approach and selection of ablation target in the NSC- aided group**

A high-density endocardial and/or epicardial bipolar EAM of the area of interest was acquired during stable sinus rhythm or right ventricle (RV) apex pacing. Electrograms (EGMs) with delayed components were classified as entrance or inner conducting channel points depending on delayed-component (near-field) precocity. At sites where hidden slow conduction (HSC)–EGMs were suspected (EGMs with >3 deflections not meeting criteria for fractionated pathological EGM identified within the scar area or in the myocardium surrounding the scar area), a double ventricular extra stimulus was delivered: response was considered positive when the local potential delayed and split from the far-field signal[17](#_ENREF_17). According to the scar dechannelling technique[18](#_ENREF_18), all the identified conducting channels entrance EGMs and HSC–EGMs were targeted for ablation, aiming at the abolition of the pathological EGMs. Each RF application lasted 30 to 60 seconds, aiming at the complete local pathological potential abolition. Backup RF applications were delivered inside the scar area when the RF delivery at the described ablation targets did not eliminate internal pathological EGMs.

**Definition of incomplete substrate ablation**

Incomplete substrate ablation was defined in the presence of one of the following scenarios: i) the persistence of residual pathological EGM at the end of the ablation, ii) the presence of any non-reachable midmyocardial septal VT substrate, or iii) epicardial non-accessibility in patients with evidence of epicardial substrate according to cardiac imaging.

**MDCT post-processing**

The segmentation process employs a well-established machine learning pipeline, consisting of two stages: the Localizer and the Segmenter. The Localizer generates a low-resolution binary mask of the left ventricle to identify its approximate location. This binary mask is then used to define a 130×130×130 mm³ volumetric Region of Interest (ROI) in the resampled CT (0.5 mm³ resolution) and provides additional spatial information for the second stage. The Segmenter produces a multi-level segmentation mask encoding the following regions: Background, Endocardium, and Epicardium. The resulting mask undergoes post-processing to eliminate any undesired artifacts or satellite regions, ensuring accurate delineation of the left ventricle structures. These refined masks are then converted to polygonal meshes representing the endocardial and epicardial surfaces, enabling the calculation of wall thickness for clinical analysis.
