# Supplementary material for: Outcomes of ventricular tachycardia ablation facilitated by pre-procedural cardiac imaging-derived scar characterization: a prospective multi-centre international registry
Source: Europace. 2025 Mar 14;27(4):euaf051. doi: 10.1093/europace/euaf051 (PMC11983391; doi:10.1093/europace/euaf051)
Supplement: euaf051_Supplementary_Data [file euaf051_supplementary_data.zip › Supplemental Tables.docx]

|  | Year | Study Design | Patients | Substrate Ablation Strategy | Procedure Time (min) | RF Time (min) | Fluoroscopy Time (min) | Procedural Adverse Event Rate | VT-free Survival |
| --- | --- | --- | --- | --- | --- | --- | --- | --- | --- |
| Jais et al. (1) | 2012 | Prospective, 2 centers | 70 (CAD, IDCM) | LAVA elimination | 148 ± 73 | 23 ± 11 | 42 ± 20 | 8.6% | 64% at 22 months |
| Di Biase et al. (2) | 2012 | Prospective, randomized multicenter | 49 vs. 43 (ICM) | Endo substrate ablation vs, scar homogenization | 216±78 vs. 288±90 | 39±17 vs. 74±21 | 32±14 vs. 38±11 | - | 53% vs. 81% at 22 months |
| Berruezo et al. (3) | 2015 | Prospective, single center | 101 (CAD, NICM) | Scar dechannelling | 227 ± 69 | 28 ± 16 | 18 ± 8 | 6.9% | 73% at 21 months |
| Aziz et al. (4) | 2019 | Prospective, single center | 120 (CAD, NICM) | Targeting of deceleration zones | 312 (240-414) | 29 (21-38) | - | 8% | 70% at 12 months |
| Fernande-Armenta et al. (5) | 2020 | Prospective, multicenter | 412 (CAD, NICM) | Scar dechannelling (SD) | 212 ± 72 | 21 ± 14 | 20 ± 12 | 6.8% | 72% at 24 months |
| Radinovic et al. (6) | 2022 | Prospective, multicenter | 309 (CAD, NICM) | LPs elimination | 218 ± 77 | 24 ± 17 | 30 ± 19 | 10% | 67% at 12 months |
| Penela et al. | 2024 | Prospective, multicenter | 171 (CAD, NICM) | SD with NSC integration | 192±72 | 16±11 | 16±10 | 5% | 74% at 18 months |

**Supplemental Table 1. Outcomes of registries using substrate-guided VT ablation strategies.**

Coronary Artery Disease; CC = Conducting Channel; EGM = Electrograms; ICM = Ischemic Cardiomyopathy; LAVA = Local Abnormal Ventricular Activity; LP = Late Potential; LVEF = Left Ventricle Ejection Fraction; RF = Radiofrequency; VT = Ventricular Tachycardia.

**Supplemental Table 2**. Predictors of ventricular tachycardia recurrence at univariate and multivariate Cox proportional hazard model.

|  | Univariate analysis | |  | Multivariate analysis | |  |  |
| --- | --- | --- | --- | --- | --- | --- | --- |
|  | Hazard ratio (95% CI) | p value |  | Hazard ratio (95% CI) | p value |  |  |
| **Age (years)** | 1.01 (0.98-1.03) | 0.57 |  |  |  |  |  |
| **Male** | 2.27 (0.31-16.65) | 0.42 |  |  |  |  |  |
| **Hypertension** | 1.35 (0.70-2.59) | 0.37 |  |  |  |  |  |
| **Dyslipidemia** | 1.01 (0.55-1.85) | 0.98 |  |  |  |  |  |
| **Type 2 diabetes** | 2.01 (0.95-3.86) | 0.12 |  |  |  |  |  |
| **COPD** | 2.29 (0.96-5.56) | 0.11 |  |  |  |  |  |
| **Atrial Fibrillation** | 1.02 (0.33-2.65) | 0.92 |  |  |  |  |  |
| **CKD** | 1.62 (0.71-3.67) | 0.25 |  |  |  |  |  |
| **LVEF (%)** | 0.97 (0.94-0.99) | **0.05** |  | 0.98 (0.95-1.01) | 0.14 |  |  |
| **ICM** | 0.95 (0.50-1.79) | 0.86 |  |  |  |  |  |
| **VT storm** | 2.26 (1.15-4.45) | **0.02** |  | 1.97 (1.01-3.93) | **0.05** |  |  |
| **Preprocedural CMR** | 1.05 (0.92-1.13) | 0.99 |  |  |  |  |  |
| **Image-guided ablation** | 1.44 (0.72-2.87) | 0.31 |  |  |  |  |  |
| **VT non-inducibility post-first ablation attempt** | 0.78 (0.46-1.54) | 0.29 |  |  |  |  |  |
| **Incomplete substrate ablation** | 0.84 (0.25-2.77) | 0.77 |  |  |  |  |  |
| **Acute procedural success** | 0.49 (0.27-0.90) | **0.02** |  | 0.53 (0.29-0.98) | **0.04** |  |  |
| **AAD assumption at follow-up** | 1.79 (1.09-3.52) | 0.13 |  |  |  |  |  |

AAD = Anti-Arrhythmic Drug; CMR = Cardiac Magnetic Resonance; COPD = Chronic Obstructive Pulmonary Disease; CKD = Chronic Kidney Disease; ICM = Ischemic Cardiopathy; LVEF = Left Ventricle Ejection Fraction;

References:

1.- Jais P, Maury P, Khairy P et al. Elimination of local abnormal ventricular activities: a new

end point for substrate modification in patients with scar-related ventricular tachycardia. Circulation

2012;125:2184-96.

2.- Di Biase L, Burkhardt JD, Lakkireddy D et al. Ablation of Stable VTs Versus Substrate

Ablation in Ischemic Cardiomyopathy: The VISTA Randomized Multicenter Trial. J Am Coll

Cardiol 2015;66:2872-2882.

3.- Berruezo A, Fernandez-Armenta J, Andreu D et al. Scar dechanneling: new method for scar-

related left ventricular tachycardia substrate ablation. Circ Arrhythm Electrophysiol 2015;8:326-36.

4.- [Targeted Ablation of Ventricular Tachycardia Guided by Wavefront Discontinuities During Sinus Rhythm: A New Functional Substrate Mapping Strategy.](https://pubmed.ncbi.nlm.nih.gov/31533463/)

Aziz Z, Shatz D, Raiman M, Upadhyay GA, Beaser AD, Besser SA, Shatz NA, Fu Z, Jiang R, Nishimura T, Liao H, Nayak HM, Tung R.Circulation. 2019 Oct 22;140(17):1383-1397.

5.- Fernandez-Armenta J, Soto-Iglesias D, Silva E et al. Safety and Outcomes of Ventricular

Tachycardia Substrate Ablation During Sinus Rhythm: A Prospective Multicenter Registry. JACC

Clin Electrophysiol 2020;6:1435-1448.

6.- Radinovic A, Peretto G, Sgarito G et al. Matching ablation endpoints to long-term outcome:

Data from the prospective multicenter Italian VT ablation Registry. JACC Clin Electrophysiol

2023;9:836-847.
