# Supplementary figures and images for: Outcomes of ventricular tachycardia ablation facilitated by pre-procedural cardiac imaging-derived scar characterization: a prospective multi-centre international registry
Source: Europace. 2025 Mar 14;27(4):euaf051. doi: 10.1093/europace/euaf051 (PMC11983391; doi:10.1093/europace/euaf051)

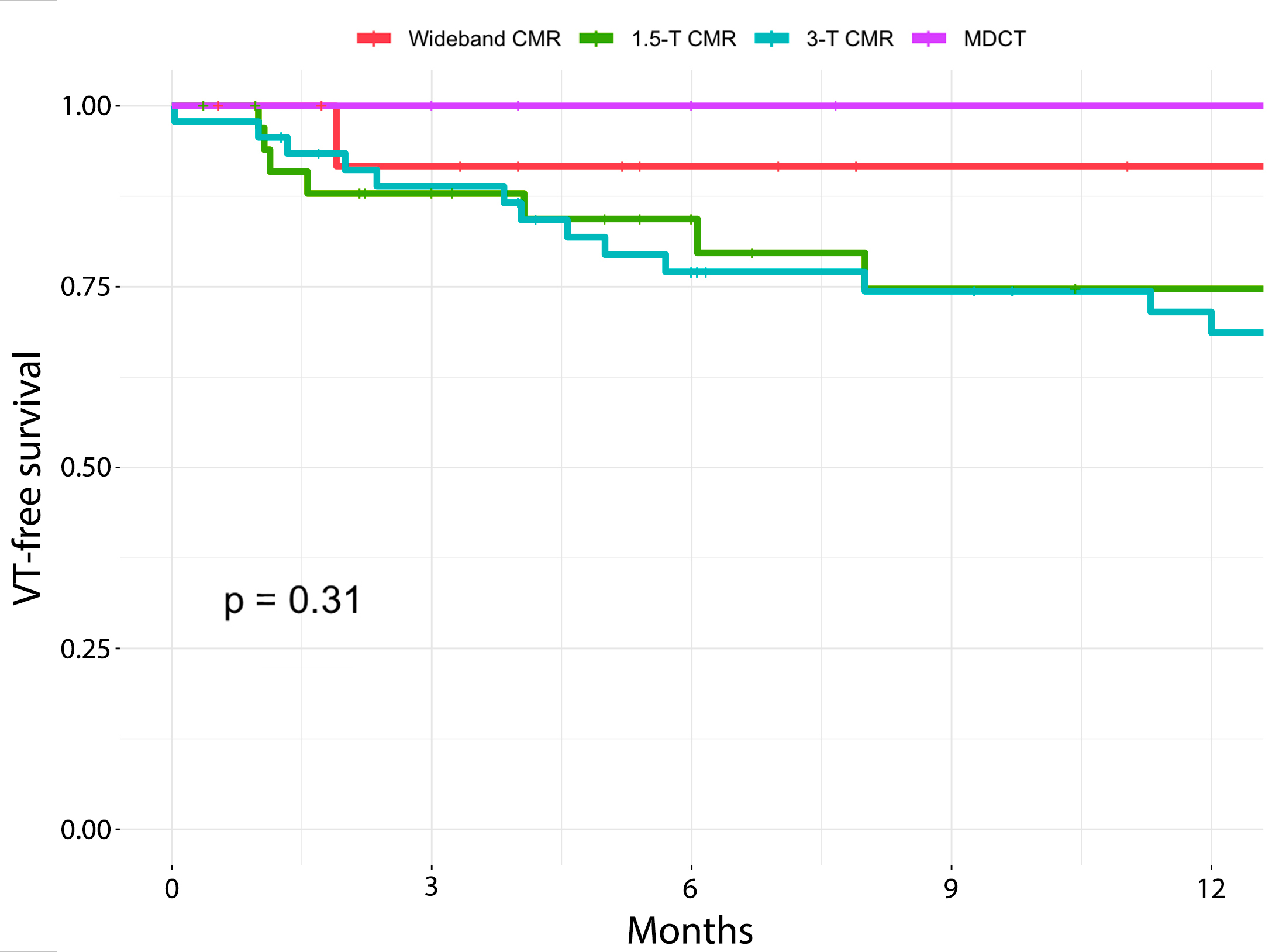

Supplement: euaf051_Supplementary_Data [file euaf051_supplementary_data.zip › Supl Figure 1.jpg]

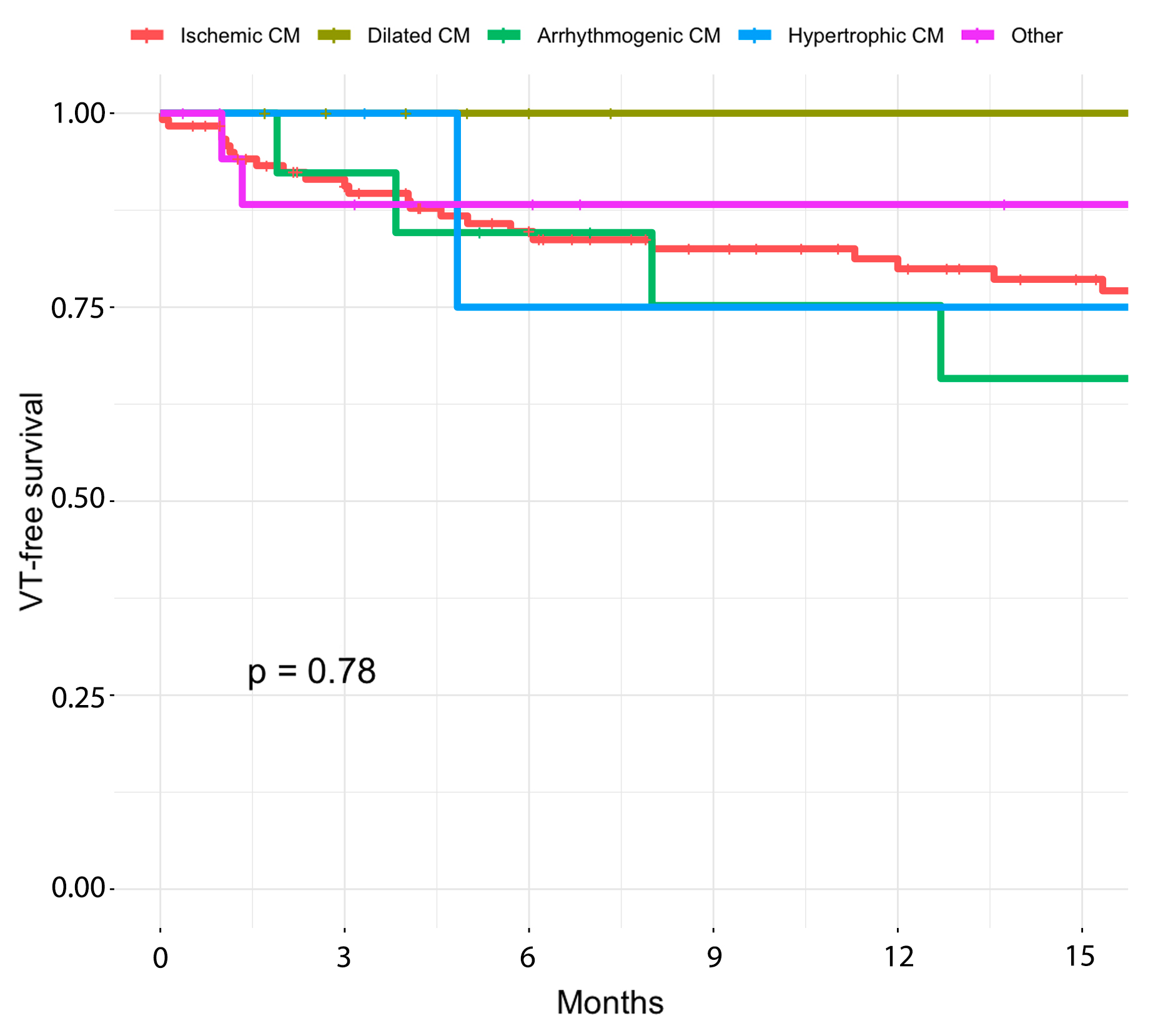

Supplement: euaf051_Supplementary_Data [file euaf051_supplementary_data.zip › Supl Figure 2.jpg]
